# POLYCYSTIC OVARY SYNDROME: ORIGINS AND IMPLICATIONS: The impact of polycystic ovary syndrome on reproductive health: a narrative review

**DOI:** 10.1530/REP-24-0485

**Published:** 2025-04-29

**Authors:** Lisa Ann Owens, Stephen Franks

**Affiliations:** ^1^ Department of Endocrinology, St James’s Hospital, Dublin, Ireland; ^2^ School of Medicine, Trinity College Dublin, Dublin, Ireland; ^3^ Institute of Reproductive & Developmental Biology, Department of Metabolism, Digestion & Reproduction, Imperial College London, London, UK

**Keywords:** polycystic ovary syndrome, fertility, pre-pregnancy care, pregnancy, anovulation, menstrual dysfunction, sexual function

## Abstract

Polycystic ovary syndrome (PCOS) is the most common endocrinopathy in women of reproductive age. The condition can have an enduring negative effect on women’s reproductive health from menarche to menopause, although its impact can vary significantly. PCOS is associated with premature pubarche and a wider age range of menarche. Diagnosis of PCOS in adolescents remains challenging. Oligo/anovulation is the most common feature of PCOS, and leads to subfertility. This may require induction of ovulation with medical therapies, such as oestrogen receptor antagonists, aromatase inhibitors or by giving exogenous follicle-stimulating hormone, which are effective for most women with PCOS. Pregnancy in women with PCOS is associated with a higher risk of complications including gestational diabetes, particularly in those with obesity. Optimisation of pre-conception health, including weight management, is recommended in order to maximise fertility potential and improve pregnancy outcomes. The other key feature of PCOS is hyperandrogenism, which may contribute to ovulatory dysfunction and results in hirsutism and persistent acne, and also negatively impacts mental health, quality of life and psychosexual function. Women with PCOS may also have a later age of menopause, although longitudinal studies are lacking. This narrative review explores the impact of PCOS on women’s reproductive health throughout their reproductive lifespan.

## Introduction

Polycystic ovary syndrome (PCOS) is the most common endocrine disorder in women of reproductive age, with an estimated global prevalence of between 10 and 13% (Alvarez-Blasco *et al.* 2006, Bozdag *et al.* 2016, Teede *et al.* 2023). PCOS is characterised by the presence of oligo- or amenorrhoea, ovarian hyperandrogenism and polycystic ovarian morphology (PCOM). It is a heterogeneous condition, which can be associated with an increased incidence of long-term health conditions including obesity, type 2 diabetes, cardiovascular disease and mental illness. PCOS can have a significant impact on a woman’s reproductive health throughout her reproductive lifespan (Fig. 2), although the impact varies significantly between women.

First, PCOS is associated with premature pubarche, and a wider age range at menarche, ranging from early menarche to primary amenorrhoea. Ovulatory dysfunction is a cardinal feature of PCOS, and most women with PCOS experience irregular menstrual cycles (Conway *et al.* 1989, Kiddy *et al.* 1990, Carmina *et al.* 2006, Azziz *et al.* 2019). Women with PCOS who have regular menstrual cycles can still have oligo-ovulation (Chang *et al.* 2005). However studies have shown that menstrual cycles can become more regular as women with PCOS age (Elting *et al.* 2000, Elting *et al.* 2003, Alsamarai *et al.* 2009).

While pregnancy rates are similar amongst younger women with and without PCOS, older women with PCOS have been found to have lower numbers of deliveries overall, lower average number of children and higher rates of infertility (West *et al.* 2014*a*,*b*). The overall health of women with PCOS should be optimised pre-conception in order to maximise fertility potential and improve pregnancy outcomes. It is recommended to assess and optimise weight, blood pressure, smoking status, alcohol intake, nutritional status, lifestyle, folate supplementation and mental and sexual health in advance of any pregnancy (Teede *et al.* 2023). Pregnancy in women with PCOS is associated with a higher risk of complications such as miscarriage, pre-eclampsia, low birth weight and gestational diabetes mellitus (GDM).

Ovulation induction (OI) may be required to achieve pregnancy in infertile women with PCOS who have oligo- or amenorrhoea. A full overview of OI and assisted reproductive technologies in PCOS is discussed below. PCOS can also have an impact on women’s psychosexual function, with studies reporting lower sexual arousal and sexual satisfaction in women with PCOS. The presence of hirsutism appears to have a negative impact on a woman’s sexual function and mental health (Karjula *et al.* 2017). Women with PCOS appear to have a later age of menopause (Minooee *et al.* 2018, Forslund *et al.* 2019, Zhu & Goodarzi 2022). The experience of women with PCOS in peri/postmenopause is poorly studied, as are the benefits and risks associated with use of menopausal hormone replacement therapy.

This narrative review gives a detailed overview of the available data on the various impacts of PCOS on women’s reproductive health throughout their life span and the current recommendations with regards to how to assess these reproductive features of PCOS. It also highlights unanswered questions and points to future research studies that are required in these important areas.

**Figure 1:**
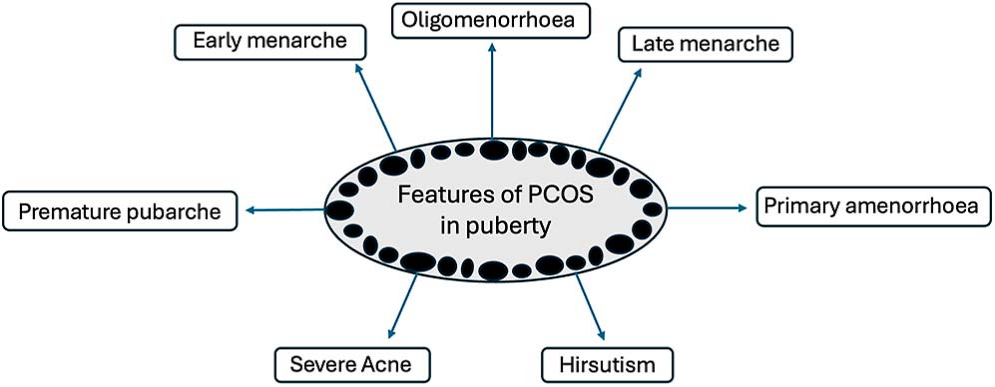
The heterogenous features and varying presentation of polycystic ovary syndrome during the pubertal transition. Premature pubarche may be an early sign of PCOS and is more common in girls who go on to be diagnosed with PCOS. Girls with PCOS appear to have a wider age range at the time of menarche compared with their peers. They can experience early menarche or late menarche and primary amenorrhea. Higher weight is associated with earlier age of menarche in girls with PCOS, and conversely those with a lower BMI have a later age of menarche. PCOS in adolescence can present as severe acne and hirsutism, as well as oligomenorrhoea.

## Puberty/age of menarche (Fig. 1)

During adolescence and puberty, there is significant overlap between the features of PCOS and what is normal for that life stage; therefore defining abnormality at this time of life remains challenging. Indeed, the most controversy surrounding the PCOS diagnostic criteria is during the pubertal transition. Irregular menstrual cycles are normal in the first year post-menarche. More than 1 but less than 3 years post-menarche, irregular cycles are defined as those lasting less than 21, or greater than 45 days. More than 3 years post-menarche, irregular cycles are defined as those lasting less than 21 or greater than 35 days, or less than eight cycles per year, or any one cycle last more than 90 days (Teede *et al.* 2023). It is essential for healthcare professionals to understand what is considered normal for pubertal stage to avoid overdiagnosis at this young age. Women within 3 years of menarche with irregular cycles alone, without hyperandrogenism, should be considered ‘at risk’ for PCOS, and re-evaluated during reassessment at the stage of reproductive maturity. It is also not recommended to use serum AMH or pelvic ultrasound to diagnose PCOS within 8 years of menarche due to significant overlap between those with and without PCOS (Teede *et al.* 2023).

Premature pubarche (development of pubic/axillary hair before the age of 8 years) may be an early sign of PCOS and is more common in girls who go on to be diagnosed with PCOS (Ibañez *et al.* 1993, Carroll *et al.* 2012). However, not all girls with PCOS will have premature pubarche and similarly not all girls who experience premature pubarche will go on to be diagnosed with PCOS. Girls with PCOS appear to have a wider age range at the time of menarche compared with their peers. They can experience early menarche (at or before 9 years of age) or late menarche and primary amenorrhoea (menarche absent age 16 years or 4 years after the onset of thelarche).

There are limited data examining what predicts age at menarche in girls with PCOS, but it appears to be driven by several opposing factors. Higher weight is associated with earlier age of menarche (Stark *et al.* 1989, Carroll *et al.* 2012). One retrospective study demonstrated that girls with PCOS and with a higher body mass index (BMI) than their peers had an earlier age of menarche, whereas girls with PCOS who have lower BMI compared to their peers had a later age of menarche (Carroll *et al.* 2012). Girls who were born at a low birth weight are also more likely to have premature pubarche and earlier menarche (Ibáñez *et al.* 2006). Girls may present with primary amenorrhoea and subsequently go on to be diagnosed with PCOS. One small, retrospective study found that girls with PCOS who have primary amenorrhoea were more likely to have features of the metabolic syndrome and higher androstenedione levels than those who were post-menarche, therefore suggesting a more severe phenotype of PCOS (Rachmiel *et al.* 2008). More work is needed to assess predictors of puberty and menarche in this cohort.

The recognition of PCOS in adolescence, while challenging, is important in order to facilitate appropriate monitoring and treatment. Studies have shown that lifestyle interventions can reduce weight and improve menstrual cycle regularity and hyperandrogenic symptoms in adolescents with PCOS (Babaei Bonab & Parvaneh 2023, Jakubowska-Kowal *et al.* 2024, Nahidi *et al.* 2024). While there is limited evidence for pharmacotherapy for adolescents with PCOS, the international guidelines also recommend considering use of metformin and combined oral contraceptives in adolescents with or at risk for PCOS (Teede *et al.* 2023).

## Irregular menstrual cycles and ovulatory dysfunction

Irregular menstrual cycles and ovulatory dysfunction are characteristic features of PCOS, and a key component of the diagnostic criteria (Rotterdam ESHRE/ASRM-Sponsored PCOS Consensus Workshop Group). Most (75–85%) women with PCOS have irregular menstrual cycles, as assessed in many retrospective case series (Conway *et al.* 1989, Kiddy *et al.* 1990, Alvarez-Blasco *et al.* 2006, Carmina *et al.* 2006, Azziz *et al.* 2019). The exact mechanisms of anovulation in PCOS remain unclear, but there is evidence for disordered follicle development from the earliest phases through to the antral stages, with persistence and assumed arrest of larger antral follicles. It is likely that these ‘arrested’ follicles comprise those that have already undergone terminal differentiation (being prematurely responsive to LH) together with ‘healthy’ follicles that have stopped growing due to suboptimal FSH stimulation but would resume growth in response to a spontaneous or induced increase in FSH (Franks *et al.* 2008, Jarrett *et al.* 2020).

The presence of irregular cycles and hyperandrogenism in women with PCOS has been shown to be associated with higher metabolic risk than those who present with either oligomenorrhoea or hyperandrogenism alone. Insulin resistance (IR) affects most women with PCOS, especially those who are overweight or obese (Dunaif *et al.* 1989, Carmina & Lobo 2004, Stepto *et al.* 2013, Cassar *et al.* 2016). Studies have shown that the severity of IR correlates positively with the time between menstrual bleeding (Brower *et al.* 2013, Hussein & Karami 2023). One study showed that 20% of women with PCOS reported vaginal bleeding intervals of fewer than 35 days in length and did not generally have overt insulin resistance, regardless of whether the cycles were ovulatory or not (Brower *et al.* 2013).

Ovulatory dysfunction can also occur in women with PCOS who have regular menstrual cycles. In one study of 316 women with PCOS, as defined by NIH 1990 criteria, 16% had eumenorrhoea (cycles every 27–34 days) but had oligo/anovulation as defined by luteal phase progesterone measurements (Chang *et al.* 2005). The 2023 international PCOS guidelines acknowledge that ovulatory dysfunction can still occur with regular cycles and recommend that, if anovulation needs to be confirmed, then luteal phase serum progesterone levels can be measured (Teede *et al.* 2023). Polymenorrhoea (more frequent cycles, occurring at intervals of <26 days) is uncommon in PCOS, occurring in only 1.5% in one study of 716 consecutive untreated patients with PCOS (Azziz *et al.* 2004).

Studies have shown that ovulatory function improves with age in women with PCOS. Menstrual frequency increases and women may actually develop normal ovulatory function and regular cycles in their 30s and 40s (Elting *et al.* 2000, Alsamarai *et al.* 2009, Carmina *et al.* 2012*a*). As women with PCOS age, androgen levels decline, and ovarian volume, follicle number and AMH levels decrease (and FSH may increase), and these markers predict a return to ovulatory cycles (Elting *et al.* 2003, Azziz *et al.* 2009, Carmina *et al.* 2012*a*,*b*). Indeed, one longitudinal study demonstrated that the phenotypic changes that women with PCOS experience as they age mean that most (75.5%) of them at the age of 50 no longer meet the diagnostic criteria for the condition (van Keizerswaard *et al.* 2022). There is further discussion around menopause in women with PCOS in section Menopause 2.7.

## Fertility

### Fecundity/pregnancy rates in women with PCOS

Given that most women with PCOS experience oligo/anovulation, it would be expected that the condition would be associated with reduced fertility/fecundity. However, one study followed 91 women with PCOS and 87 controls and found that 86.7% of PCOS patients and 91.6% of controls had given birth to at least one child (Hudecova *et al.* 2009). Most (73.6%) of the cohort of women with PCOS reported spontaneous conception. A Swedish longitudinal study followed 27 women with PCOS and 94 control women between 1998 and 2016 and found no difference in parity between groups (Forslund *et al.* 2019). Similarly, the prospective North Finland Birth Cohort 1986 study found that women reporting menstrual irregularity at the age of 16 years became pregnant at least once, and had at least one delivery or one miscarriage by age 26, as often as women with regular menstruation, and had similar mean pregnancy, delivery and spontaneous abortion rates by the age of 26 years (West *et al.* 2014*a*). However, women with irregular cycles at the age of 16 or a diagnosis of PCOS did report more fertility problems. The North Finland Birth Cohort 1966 study, however, looked at women age 44 and found that although women with hirsutism and oligomenorrhoea delivered one child as often as asymptomatic women and were of a similar age at first delivery and had similar incidence of miscarriage, they did have fewer deliveries overall and slightly smaller family size (mean (SD) = 1.9 (0.8) versus 2.4 (1.4)) (West *et al.* 2014*b*). In this cohort, women with PCOS symptoms had been treated more often for infertility than asymptomatic women in this study also. In addition, normal weight and obese women with PCOS had significantly fewer deliveries than obese women without PCOS. Obese women with PCOS had the fewest number of deliveries overall (West *et al.* 2014*b*).

### Pre-conception care

Preconception care is defined by the 2023 international PCOS guidelines as a ‘set of interventions that aim to identify and modify biomedical, behavioural and social risks to a woman’s health or pregnancy outcome through prevention and management, emphasising those factors that must be acted on before conception or early in pregnancy to have maximal impact’ (Teede *et al.* 2023). Pregnancy in women with PCOS is complicated by an increased incidence of gestational diabetes, hypertension, pre-eclampsia, miscarriage, pre-term birth, ovarian hyperstimulation syndrome, perinatal anxiety and depression; therefore, pre-pregnancy assessment and advice about health optimisation is vital. Pre-conception care for women with PCOS should, ideally, be offered in advance of them trying to conceive, to allow time for modifiable risk factors to be addressed. A meta-analysis completed for the 2023 international PCOS guidelines looked at 16 studies and found that women with PCOS who were in the healthy weight category had a higher clinical (1.54 (1.09, 2.18)) pregnancy rate and live birth rate (1.39 (1.17, 1.65)) than those with PCOS who were in the higher weight categories (Teede *et al.* 2023). They also assessed 11 studies and found that those with PCOS in the normal weight category had a higher ovulation rate per patient and per cycle and lower miscarriage rate (0.64 (0.59, 0.71)) compared to women in the higher BMI category. Therefore, it is recommended that women with PCOS should be counselled in advance of pregnancy on the adverse impact of excess weight on pregnancy outcome. There are, however, no prospective studies assessing optimal proportion of weight loss or interventions for weight loss, or optimal models of care delivery in general for this young cohort pre-pregnancy. Future studies should address these gaps. Women should also be counselled early on about the impact of advanced maternal age on conception and pregnancy outcome. One study showed that between age 40 and 45, pregnancy and live birth rates with *in vitro* fertilisation (IVF) in women with PCOS decline and the rate of miscarriage rate increases, at a similar rate, despite having a higher oocyte yield (Kalra *et al.* 2013).

### Ovulation induction and assisted reproductive technology (ART)

Methods of OI include medications such as letrozole, clomiphene citrate (CC), metformin, gonadotrophins and laparoscopic ovarian surgery (LOS). ‘Letrozole is an oral, highly selective, reversible aromatase inhibitor that blocks aromatase activity and prevents androgen conversion to oestrogens, thus increasing FSH secretion by removing oestrogen-mediated negative feedback. CC is a mixed agonist–antagonist to oestrogens that stimulates gonadotropin release by binding to oestrogen receptors in hypothalamus, and, like letrozole, blocking the negative feedback of oestradiol. The mechanism of action of metformin, a biguanide insulin-sensitising agent, on reproductive function in OI is unclear, although weight loss may play a part in improving menstrual cyclicity.’

While letrozole and CC are both effective at OI, letrozole, an aromatase inhibitor, is now considered the first-line pharmacological agent for OI in infertile women with PCOS. Meta-analysis of 11 randomised-controlled trials (RCTs) demonstrated that it is superior to CC for improving ovulation rate, pregnancy and live birth rate (Teede *et al.* 2023). There is no difference between the two in terms of miscarriage rate or multiple pregnancy rate. A meta-analysis of six studies showed that metformin was superior to placebo in terms of increased live birth, clinical pregnancy and pregnancy rates, but was much less effective than letrozole or CC in terms of ovulation rate, pregnancy and live birth rate (Teede *et al.* 2023). However, use of metformin involves less monitoring and has a lower rate of multiple pregnancies, and therefore it should be considered for some women, especially younger women or those with limited access to specialist fertility services.

Gonadotrophin therapy is used second-line in women with PCOS who have not conceived with first-line therapy. Careful monitoring of follicular development by ultrasound is required to achieve a single dominant follicle and a low dose step-up protocol is recommended to reduce multiple pregnancy and ovarian hyperstimulation rate. One retrospective study assessed the safety and efficacy of induction of ovulation with gonadotrophin therapy in 364 women with PCOS. Women had been treated with human menopausal gonadotropin, purified urinary FSH or recombinant human FSH. This study found that the proportion of ovulatory cycles that women had was high (83%) and the pregnancy rate was more than 90% within six cycles of treatment. One quarter of cycles had to be abandoned due to multiple follicle development and 4% of women had multiple pregnancies. The median threshold dose of gonadotrophin required to induce ovulation was 75 IU/day (White *et al.* 2018). Meta-analyses have actually demonstrated that gonadotrophins are superior to CC and LOS for ovulation, pregnancy and live birth rates (Teede *et al.* 2023). However, consideration should be given to cost, expertise required, degree of intensive ultrasound monitoring required and risks of multiple pregnancies. There are no differences in outcomes between the various gonadotrophin preparations.

LOS is a keyhole surgical procedure, in which a diathermy instrument is used to puncture the ovarian cortex, followed by the delivery of an electrical current to the ovarian stroma. Studies and meta-analyses show that rates of ovarian hyperstimulation syndrome and multiple pregnancies appear lower than with other options (Eftekhar *et al.* 2016, Teede *et al.* 2023). Currently, the 2023 international PCOS guidelines state that it could be used as a second-line therapy for anovulatory infertile women with PCOS with resistance to CC or letrozole and no other cause of infertility; however, consideration should be given to the cost involved, expertise required and operative risks (Teede *et al.* 2023). Meta-analysis of three studies in women with PCOS and CC resistance showed that gonadotrophins were superior to LOS for live birth rates OR 2.21 (95% CI 1.32, 3.71) but had a much higher rate of multiple pregnancy OR 5.10 (95% CI 1.39, 18.68), with no difference in other outcomes (Teede *et al.* 2023). Further randomised studies, which clarify the role of LOS in comparison with other treatments, are required.

Weight loss, either by lifestyle modification, bariatric surgery or pharmacological therapies, should also be considered in the OI algorithm. Weight loss of 5–10% with lifestyle interventions can improve ovulation rates (Clark *et al.* 1998, Tolino *et al.* 2005, Harrison *et al.* 2011). Bariatric surgery has been found to be more effective than medical care in improving the rate of spontaneous ovulation in women with PCOS (Samarasinghe *et al.* 2024). Many anti-obesity pharmacological agents have been proven to be effective in the general population, but have not been well-studied in women with PCOS, nor in pregnancy; therefore, it is recommended currently that they are only used in research settings (Teede *et al.* 2023).

ART may also be required in women with PCOS, either in those in whom OI has not been effective, or those who have other causes of infertility (e.g. male factor or tubal issues). It is recommended that IVF is offered to women with PCOS and anovulatory infertility if first- or second-line OI therapies have failed. Women with PCOS undergoing IVF are at a higher risk of OHSS. Gonadotrophin-releasing hormone (GnRH) antagonist protocol and GnRH agonist long protocol are associated with similar clinical pregnancy and live birth rates in women with PCOS. Protocols using GnRH antagonist are preferred as they enable the use of an agonist trigger of ovulation, reducing the risk of OHSS and allowing the freezing of all embryos, if required. Metformin therapy can be used as an adjunctive therapy in women undergoing GnRH agonist long protocol, as it may reduce the risk of OHSS and miscarriage (Teede *et al.* 2023). It is recommended that in those at high risk of OHSS, GnRH agonists are used to trigger ovulation and embryos are frozen for use in a future cycle (Teede *et al.* 2023).

## Pregnancy

Women with PCOS have higher risk pregnancies, and it is recommended that PCOS status is identified in women antenatally, and that appropriate monitoring for complications should be undertaken, including blood pressure monitoring and diabetes screening. Pregnancy risks in women with PCOS include excessive gestational weight gain, higher rates of GDM, pregnancy induced hypertension, pre-eclampsia, miscarriage, low birth weight, growth restriction and caesarean section delivery. It is recommended that women with PCOS be counselled about lifestyle interventions to minimise gestational weight gain (Teede *et al.* 2023). They should also be screened for GDM prenatally and early in pregnancy and undergo routine screening at 24–28 weeks’ gestation. Symptoms of depression and anxiety are more common in pregnant women with PCOS compared to pregnant women without PCOS (Floyd *et al.* 2024). There are no studies addressing the optimal screening and management of perinatal psychiatric morbidity in this cohort. A more detailed review of pregnancy and offspring outcomes in this cohort is beyond the scope of this review.

## Psychosexual function

Psychosexual dysfunction encompasses sexual problems or difficulties that are psychological in nature, based on cognitions and/or emotions such as depression, anxiety, low self-esteem and negative body image (Watson & Davies 1997). Women with PCOS are known to experience psychological disturbance more than their peers. In addition, in studies in the general female population, therapies used in PCOS, such as oral contraceptives, can also affect psychosexual function (Clayton *et al.* 2018). Studies show that women with PCOS score significantly lower on psychosexual function questionnaires. However, the clinical significance of this is unclear as most studies have not measured distress scores, which are required to meet the criteria for psychosexual dysfunction. One case control study in Iranian women found more sexual pain and sexual distress in women with PCOS than controls (Tahmasbi *et al.* 2024), and another in Dutch women found lower sexual function and higher levels of sexual distress in women with PCOS than controls (Pastoor *et al.* 2023). The 2023 international PCOS guidelines performed a meta-analysis on this topic as part of the guideline development process (Teede *et al.* 2023). Their pooled analysis demonstrated that ‘those with PCOS had lower total sexual function in 17 studies (OR −2.42 (−3.26, −1.58)), sexual arousal in 14 studies (−0.36 (−0.59, −0.13)), lubrication (−0.47 (−0.75, −0.20)), orgasm (−0.35 (−0.52, −0.17)) and satisfaction’. Women with PCOS had reduced sex life satisfaction and perceptions of sexual attractiveness. The presence of hirsutism had a negative impact on sexuality and women had difficulty with social engagement due to their appearance. No differences were found in the reported frequency of pain during intercourse, sexual desire, thoughts and fantasies, or the importance of sexual satisfaction between women with PCOS and control women. The guideline recommends that healthcare professionals should consider the factors that can influence psychosexual function in PCOS and consider the screening and assessment of sexual function in order to limit the negative impact of PCOS.

## Menopause

There is a lack of longitudinal studies addressing the long-term natural history of PCOS and the manifestations of PCOS after menopause. With ageing, changes occur in all elements of the Rotterdam diagnostic criteria. This can pose diagnostic challenges for women with delayed diagnoses up to that point. Menstrual cycles become more regular in older women with PCOS (Elting *et al.* 2000, Vulpoi *et al.* 2007, Brown *et al.* 2011). Women’s follicle number and ovarian volume decrease longitudinally as they age, but the decrease in ovarian volume is less pronounced in women with PCOS. Ageing women with PCOS and irregular cycles have a higher follicle count than those with PCOS and regular cycles (Elting *et al.* 2003). Postmenopausal women with PCOS still report more hirsutism than controls (Schmidt *et al.* 2011, Forslund *et al.* 2021). Case–control studies have shown that postmenopausal women with PCOS have higher androgen (testosterone, free androgen index, 17 hydroxyprogesterone, androstenedione and DHEAS) and lower sex hormone-binding globulin levels than women without PCOS (Markopoulos *et al.* 2011, Schmidt *et al.* 2011). Another longitudinal study in Danish women only found a significant difference in DHEAS and androstenedione levels in women with and without PCOS above the age of 50 (Pinola *et al.* 2015). A study from Gothenburg of women over 80 found that they were still more hirsute, but androgen levels were similar (Forslund *et al.* 2021). It is worth noting, although androgen assays can be unreliable with poor precision in the low levels recorded in postmenopausal women (Legro *et al.* 2010).

Women with PCOS may have a later age of menopause than women without PCOS, although longitudinal data on age of menopause are lacking. Two studies tracked AMH levels in women with and without PCOS and both estimated an approximate 2 year delay in the age of menopause (Tehrani *et al.* 2010, Minooee *et al.* 2018). Their estimated ages at menopause were 51 (95% CI, 34–81) and 51.4 (95% CI 45–59) years versus 49 (95% CI, 38–63) and 49.7 (95% CI 45–55) years in PCOS cases and controls, respectively. In another study, women who self-reported having PCOS were found to have a later age of menopause compared to women with no history of PCOS (HR 0.44, 95% CI 0.28–0.71) (Li *et al.* 2016). One Swedish study that re-examined women in 2016 that were diagnosed with PCOS in 1998 and compared them with randomly selected women found that women with PCOS reached menopause 4 years later (53.3 ± 2.2 years vs 49.3 ± 3.5 years) and had lower serum follicle-stimulating hormone compared with age-matched controls (Forslund *et al.* 2019).

Menopausal hormone therapy (MHT) in women in their early postmenopausal years (<60 years) has been shown to reduce the incidence of cardiovascular disease (CVD) and all-cause mortality (Grodstein & Stampfer 1995, Grodstein & Stampfer 1998, Salpeter *et al.* 2004). CVD is the number one cause of death in postmenopausal women in the western world (Mozaffarian *et al.* 2016). Oral oestrogens also improve glucose metabolism and reduce the risk of type 2 diabetes and improving lipid profiles, including in women with a history of menstrual disorders (Li *et al.* 2021, Miller *et al.* 1995, Espeland *et al.* 1998). Women with PCOS have an increased incidence of CVD and T2DM (Cibula *et al.* 2000), therefore may theoretically have more incremental benefit from MHT use. There have, however, been no prospective trials which address the benefits and risks of MHT in women with PCOS.

The experiences of women with PCOS in the perimenopause are also poorly studied. Obese women are more likely to experience vasomotor symptoms of menopause, and the severity is proportional to changes in weight and BMI (Gold *et al.* 2006, Thurston *et al.* 2009). Women with PCOS are more likely to have obesity; however, in PCOS excess androgens are aromatised to oestrogens, which in theory could protect against oestrogen deficiency symptoms. The frequency and severity of menopausal symptoms in women with PCOS has not been studied.

## Conclusions/future perspectives

**Figure 2:**
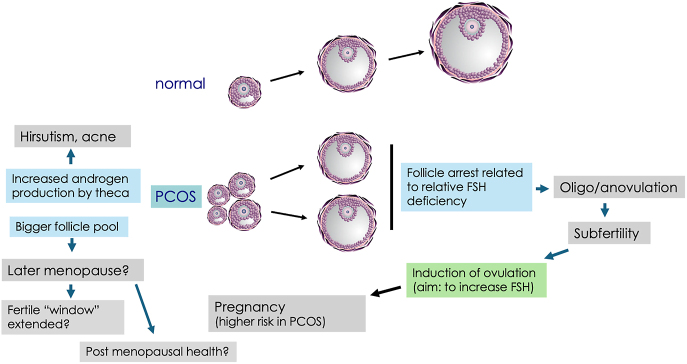
The impact of anovulation and hyperandrogenism on reproductive health in women with PCOS. Anovulation in women with PCOS is characterised by arrest of antral follicles at 5-8mm which is associated with serum FSH concentrations that, whilst in the normal range, do not reach the threshold for maturation that are characteristic of those observed in the normal early follicular phase. Induction of ovulation can be achieved by raising endogenous FSH (by an oestrogen receptor antagonist or aromatase inhibitor) or by giving exogenous FSH. Pregnancy in women with PCOS is associated with a higher risk of complications including gestational diabetes, particularly in obese women with the syndrome. The larger follicle pool may result in prolongation of fertile life and later age of menopause, but this remains to be established by further studies, as is the impact of reproductive health post menopauseHyperandrogenism results in hirsutism and persistent acne which may also impact mental health, quality of life and psychosexual function.

Polycystic ovary syndrome can impact reproductive health throughout a woman’s lifespan, from menarche through conception and pregnancy to menopause, as summarised in Fig. 2 . There are many unanswered questions with regards to the reproductive health of women with PCOS, including those focused on the optimal diagnosis and management of reproductive health issues. Future prospective research studies should aim to address the following important questions, in a list which is, by no means, exhaustive:

- How can we better identify and manage PCOS in young girls and teenagers?
- What is the optimal model of care for women with PCOS before conception?
- What is the optimal degree of weight loss in women with PCOS before pregnancy?
- What interventions should be used for weight loss in women with PCOS pre-pregnancy?
- What interventions reduce psychosexual distress in women with PCOS?
- Do women with PCOS experience more/less menopausal symptoms when compared with BMI matched controls?
- Does MHT improve CVD risk factors or incidence in women with PCOS?

## Declaration of interest

S Franks is an associate editor of *Reproduction*. S Franks was not involved in the review or editorial process for this paper, on which he is listed as an author. The authors have no conflicts of interest to declare.

## Funding

This work did not receive any specific grant from any funding agency in the public, commercial or not-for-profit sector.
